# In Situ Proximity Ligation Assays Indicate That Hemochromatosis Proteins Hfe and Transferrin Receptor 2 (Tfr2) Do Not Interact

**DOI:** 10.1371/journal.pone.0077267

**Published:** 2013-10-14

**Authors:** Gautam Rishi, Emily M. Crampton, Daniel F. Wallace, V. Nathan Subramaniam

**Affiliations:** 1 The Membrane Transport Laboratory, the Queensland Institute of Medical Research, Queensland, Australia; 2 Liver Research Centre, School of Medicine, University of Queensland, Brisbane, Queensland, Australia; University of Navarra School of Medicine and Center for Applied Medical Research (CIMA), Spain

## Abstract

The hemochromatosis associated proteins HFE and Transferrin Receptor 2 (TFR2) have been shown to be important for the proper regulation of hepcidin. A number of *in vitro* studies using transient overexpression systems have suggested that an interaction between HFE and TFR2 is required for the regulation of hepcidin. This model of iron sensing which centers upon the requirement for an interaction between HFE and TFR2 has recently been questioned with *in vivo* studies in mice from our laboratory and others which suggest that Hfe and Tfr2 can regulate hepcidin independently of each other. To re-examine the postulated interaction between Hfe and Tfr2 we developed a novel expression system in which both proteins are stably co-expressed and used the proximity ligation assay to examine the interactions between Hfe, Tfr1 and Tfr2 at a cellular level. We were able to detect the previously described interaction between Hfe and Tfr1, and heterodimers between Tfr1 and Tfr2; however no interaction between Hfe and Tfr2 was observed in our system. The results from this study indicate that Hfe and Tfr2 do not interact with each other when they are stably expressed at similar levels. Furthermore, these results support *in vivo* studies which suggest that Hfe and Tfr2 can independently regulate hepcidin.

## Introduction

Mutations in HFE and transferrin receptor 2 (TFR2) cause hereditary hemochromatosis [1,2], which is characterized by an inappropriate hepcidin (*HAMP*) expression relative to body iron levels [3]. Hepcidin, a 25 amino acid antimicrobial peptide is a negative regulator of iron stores; in response to increased iron levels in the serum, hepatic hepcidin expression is increased which then relays a signal to the sites of iron absorption (duodenal enterocytes), recycling (macrophages) and storage (hepatocytes) resulting in a decrease in the release of iron from these tissues into the circulation. 

HFE has been shown to interact with transferrin receptor 1 (TFR1) [4–6]. TFR1 is a ubiquitously expressed transmembrane receptor responsible for the uptake of transferrin (Tf) bound iron. One model of iron sensing suggests that HFE and TFR2 are required for the proper regulation of hepcidin synthesis. In this model when TFR1 bound to iron saturated transferrin (holo-Tf) is internalized, HFE is released and made available for binding to TFR2. The interaction of HFE and TFR2 is then thought to initiate a signalling cascade to regulate hepcidin. Initial studies using extracellular domains of HFE and TFR2 failed to detect an interaction between the two molecules [7]. Subsequent studies using transient overexpression systems, however, identified an interaction between HFE and TFR2 [8–10] and suggested that this interaction is required for the regulation of hepcidin [10]. In a recent study using the human hepatoma cell line (HuH-7) and transient expression systems it was shown that HFE, TFR2 and hemojuvelin (HJV) form a complex on the membrane, and the formation of this complex is required for the regulation of hepcidin [11]. 

In a recent study using transgenic mice expressing myc-tagged Hfe, no interaction was detected between the myc-tagged Hfe and Tfr2 [12]. In addition, hepatocyte specific expression of myc-tagged Hfe reduced iron stores and induced hepcidin synthesis in Tfr2 mutant mice. These results suggest that Tfr2 is not required for Hfe-mediated hepcidin synthesis [12]. In a time-course study in subjects with HFE and TFR2 mutations, hepcidin levels were measured after administration of oral iron [13]. It was shown that the patients with mutations in TFR2 did not respond to increases in transferrin saturation whereas there was a minimal hepcidin response in patients with HFE mutations under the same conditions [13]. The hepcidin response in patients with HFE mutations suggests that TFR2 can regulate hepcidin independently of HFE. 

The comparison of *Hfe* ^*-/-*^ *, Tfr2* ^*-/-*^ and double knockout mice (*Hfe* ^*-/-*^ *Tfr2* ^*-/-*^) [14] shows that there is a gradation in hepatic iron overload, with the hepatic iron concentration and serum transferrin saturation increasing from WT<Hfe^-/-^<Tfr2^-/-^< Hfe^-/-^ Tfr2^-/-^. It was also shown that there is a gradation in the levels of hepatic hepcidin relative to body iron stores in the following order WT>Hfe^-/-^>Tfr2^-/-^>Hfe^-/-^ Tfr2^-/-^, indicating that both Hfe and Tfr2 contribute to the sensing of body iron stores. In agreement with this, subjects with mutations in both *TFR2* and *HFE* present with a more severe form of disease compared to patients with either *TFR2* or *HFE* mutations alone, with a phenotype similar to juvenile hemochromatosis [15]. These observations suggest that HFE and TFR2 do not need to interact with each other to mediate a hepcidin response, prompting us to re-examine the postulated interaction between Hfe and Tfr2. 

To circumvent potential artefact issues associated with transient overexpression we used a novel co-expression system, in which FLAG-tagged Hfe and myc-tagged Tfr2 are stably expressed under the same promoter. Importantly, the relative levels of Hfe and Tfr2 are similar, unlike the previous studies where either of the two proteins was transiently over expressed. The expression and cellular localisation of Hfe, Tfr1 and Tfr2 were determined by immunoblotting and immunofluorescence. The interactions between Hfe, Tfr1 and Tfr2 were examined at a cellular level by the use of a recently developed commercial assay (Duolink^TM^) based on the principle of proximity ligation. Our results show that stably co-expressed Hfe and Tfr2 do not interact. We were able to identify previously reported interactions between Hfe and Tfr1 and the formation of heterodimers between Tfr1 and Tfr2. These results were confirmed using the conventional co-immunoprecipitation approach.

## Materials and Methods

### a: Generation of the plasmids and stable expression

The mouse *Hfe* (*mHfe*) coding sequence minus the signal peptide was amplified from mouse liver cDNA using the following primers: FP2-mHfe-Mlu CCGACGCGTGCACTGCCACCGCGT and RP2-mHfe-Mlu CCGACGCGTTCACTCACAGTCTGT and cloned into the MluI site of the pEFIRES-FLAG-S plasmid to create a bicistronic construct containing the IL-3 signal peptide fused to amino-terminally FLAG-tagged *mHfe* with the puromycin resistance gene (pac) following an internal ribosome entry site (IRES). This construct, pEFIRES-FLAG-*mHfe* and another bicistronic construct encoding amino-terminally double myc-tagged mouse transferrin receptor 2 (*mTfr2*), pEFIRES-NH2-Dmyc-*mTfr2* or pEFIRES-NH2-Dmyc-*mTfr2* ^Y245X^ (construction described in [16] were used to create a tricistronic construct encoding the *mHfe*, *mTfr2* (wild type or mutant) and pac genes each separated by an IRES sequence. The FLAG-tagged *mHfe* gene was PCR amplified along with the downstream IRES sequence from the pEFIRES-FLAG-*mHfe* plasmid using the following primers: 5’ IL-3-SP-IRES-NheI TAGGCTAGCACAATGGTTCTTG and 3’ IRES-NheI CATGCTAGCATCGTGTTTTTCAAAGGA and cloned into the NheI site of the pEFIRES-NH2-Dmyc-*mTfr2* plasmid upstream of the double-myc-tagged *mTfr2* gene. 

### b: Transfections

The mouse hepatoma cell line Hepa 1-6 was obtained from ATCC (CRL-1830; American Type Culture Collection, Manassas, VA) and cultured in DMEM with 10% fetal calf serum (FCS) in 25 cm^2^ flasks. Transfections were performed using Lipofectamine 2000 reagent according to the manufacturer’s instructions (Invitrogen, Mulgrave, Victoria, Australia). Plasmid DNA (10 μg) was complexed with 25 μl Lipofectamine 2000 reagent. The complexes were added to the cells and incubated overnight. Stably transfected cell lines were isolated by selection with 5 ug/ml puromycin for 1 day and were then stably maintained in 1 ug/ml of the antibiotic. Expression of transfectants was conﬁrmed by immunoblot analysis and immunofluorescence analysis. In the experiments involving treatment of the cells, Hepa1-6 cells expressing wild type Hfe and Tfr2 were incubated with either apo-transferrin (apo-Tf) or holo-transferrin (holo-Tf) (Sigma, Sydney, NSW, Australia) (2mg/ml) for 24 hours. The apo and holo-Tf was prepared in DMEM with 10% FCS and 1µg/ml puromycin.

### c: Western blotting

Samples were separated by 12% SDS-PAGE, transferred onto Hybond-C membrane, and blocked in 10% skim milk powder-0.1% Tween 20 in TBS (Tris-buffered saline; blocking buffer) at room temperature for 2 hours and incubated with rabbit anti-Tfr2 [17] (1µg/ml) for 2 hours at room temperature or mouse anti-FLAG M2 (1:2000; Sigma) in blocking buffer overnight at 4°C. Blots were washed extensively with 0.1% Tween 20 in TBS, and then incubated with anti-rabbit or anti-mouse IgG horseradish peroxidise (Invitrogen) for 1 h at room temperature. Lumina Forte Millipore chemiluminescent substrate (Millipore, Kilsyth, Victoria, Australia) was applied for 5 min to the blot, which was then exposed to ﬁlm (Fujiﬁlm, Brookvale, NSW, Australia). The blots were stripped with 50 mM Tris HCl pH 6.8, 1% SDS, 0.7% ß-mercaptoethanol at 50°C for 30 min, and then washed with 0.1% Tween 20 in TBS and blocked before incubating with rabbit anti-actin (Sigma) (1:3000) or mouse anti-Tfr1 (Invitrogen) (1:1500) in blocking buffer, washed with 0.1% Tween 20 in TBS, incubated with anti-mouse or anti-rabbit horseradish peroxidise (for 1 h at room temperature and then incubated with Lumina Forte Millipore chemiluminescent substrate (Millipore) for 5 min. The blot was then exposed to ﬁlm. For the immunoblots for immunoprecipitation experiments, anti-mouse or anti-rabbit light chain IgG secondary antibodies (Jackson Immunoresearch Inc, PA, USA) were used at 1:5000 for 1 hour at room temperature.

### d: Immunofluorescence and Confocal Microscopy

Cells were seeded on collagen coated glass coverslips and grown to a confluence of 75-80%. After washing with PBSCM (PBS, 1mMCaCl_2,_ 1mM MgCl_2_) three times the cells were fixed with cold 3% paraformaldehyde (PFA) for 15 minutes at room temperature (RT). The fixed cells were then washed with 50mM NH_4_Cl to quench the PFA followed by a PBSCM wash and permeabilized with 0.1% saponin in PBSCM for 15 minutes at RT and incubated with primary antibodies: rabbit anti-Tfr2 (1µg/ml), mouse anti-Tfr1 (1:500), mouse anti-FLAG M2 (1:1000) or rabbit anti-Tfr1 (Abcam, Cambridge, UK) (1:100) diluted in fluorescence dilution buffer (FDB) (5% fetal calf serum, 5% normal donkey serum, 2% bovine serum albumin in PBSCM, pH7.6) for 2 hours at RT. After washing 3 times in 0.1% saponin/PBSCM the cells were incubated with donkey anti-mouse Alexa488 and donkey anti-rabbit Alexa594 (Invitrogen) for 1 hour at RT. After washing with 0.1% saponin/PBSCM the cover slips were then mounted using Prolong Gold anti-fade with DAPI (Invitrogen). The imaging and visualisation of the fluorescently stained cells was performed using the Nikon C2 confocal microscope using a 63X oil immersion objective. We used NIS Elements software for the acquisition and processing of the images.

### e: Co-Immunoprecipitation (Co-IP)

The cells were cultured to confluence in 25 cm^2^ flasks and then lysed in an extraction buffer (200mM Tris pH8.0, 100mM NaCl, 1mM EDTA, 10% glycerol, 1mM sodium orthovanadate, 1mM sodium fluoride, 1mM sodium pyrophosphate, 2mM Phenylmethylsufonylfluoride, DNAase (1:1000), Protease Inhibitor Cocktail (1:100; Sigma) and 20 % NP 40). To perform a Co-IP with Hfe, anti-DYKDDDDK beads were used (Clontech Laboratories Inc. CA, USA). All the other antibodies used for immunoprecipitation were crosslinked with protein A/G beads (Roche, Dee Why, NSW, Australia) using dimethyl pimelimidate (Sigma). After pre-clearing with a mixture of anti-rabbit and anti-mouse IgG agarose (Sigma), 1mg of protein was incubated with anti-DYKDDDDK, anti-Tfr2, anti-rabbit IgG or anti-mouse IgG beads overnight at 4°C. The beads were then washed with 0.5% NP-40, 150mM sodium chloride, 50mM Tris pH 8.0, 5mM EDTA (NET) buffer without the detergent and resuspended in 1X sample buffer containing 5 % β-mercaptoethanol and stored at -20°C till further use.

### f: Proximity Ligation Assays (PLA, ‘In Cell’ Co-IP)

The mouse/rabbit red starter Duolink kit (Olink, Uppsala, Sweden) was used for this experiment. Hepa1-6 cells stably expressing FLAG-tagged Hfe and myc-tagged Tfr2 were seeded at 15x10^3^ cells per well in a 16-well chamber slide (Thermofisher, Scoresby, Victoria, Australia). The cells were fixed and permeabilized as described above for immunofluorescence studies. After permeabilization the cells were incubated in the blocking buffer (provided with the kit) overnight at 37°C in a humidified chamber. The following day the cells were incubated with the primary antibodies diluted in the antibody diluents for 2 hours at room temperature (used at the same concentrations as in immunofluorescence studies). For the rest of the protocol the manufacturer’s instructions were followed. Briefly, the cells were washed in Buffer A (supplied with the kit) 3 times for 15 minutes and incubated with the PLA probes for one hour at 37°C in a humid chamber. This was followed by a 10 minute wash and a 5 minute wash in Buffer A. The ligation reaction was carried out at 37°C for one hour in a humid chamber followed by a 10 and 5 minute wash in Buffer A. The cells were then incubated with the amplification mix for two hours at 37°C in a darkened humidified chamber. After washing with 1x Buffer B (supplied with the kit) for 10 minutes followed by a 1 minute wash with 0.01X buffer B the cells were mounted using the mounting media supplied with the kit. 

## Results

### a: Stable co-expression of Hfe and Tfr2 in Hepa 1-6 cells does not affect their localization

**Figure 1:**
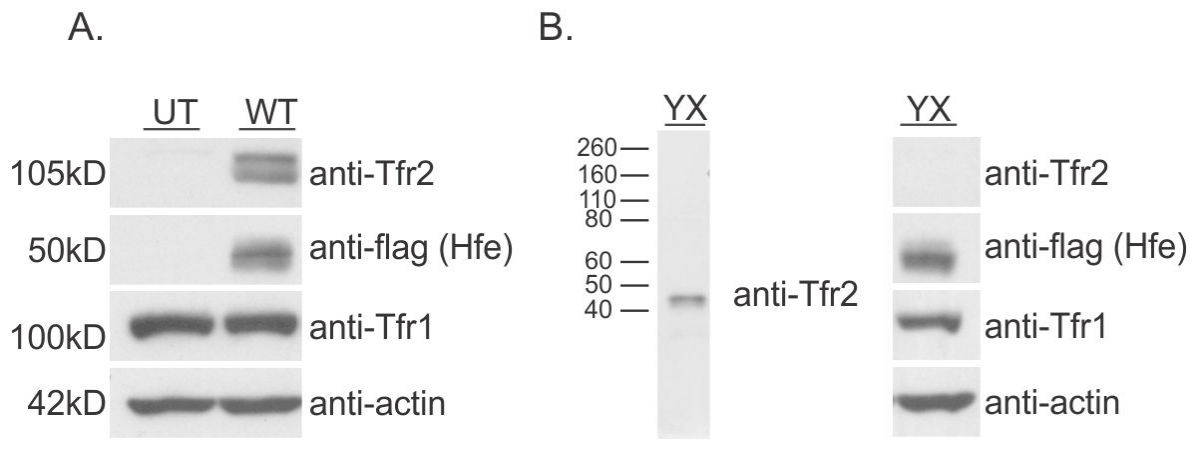
Analysis of expression of Hfe, Tfr1 and Tfr2 in transfected Hepa 1-6 cells. Hepa1-6 cells stably co-expressing FLAG-tagged Hfe and myc-tagged Tfr2 (WT), or truncated Tfr2 (YX) were analysed by Western blotting. (A) Immunoblot showing the relative expression levels of Tfr1, Tfr2 and Hfe in untransfected Hepa1-6 cells (UT), Hepa1-6 cells transfected with a vector to co-express Hfe and WT Tfr2. (B) Immunoblot showing the expression of FLAG-tagged Hfe, Tfr1 and truncated YX Tfr2 (~45kD). β-actin was used as a loading control in all the immunoblots.

In order to determine the interactions between Hfe, Tfr1 and Tfr2, Hepa 1-6 cells stably expressing N-terminal myc-tagged wild type (WT) Tfr2 or Y245X (YX) mutant Tfr2 alone or with FLAG-tagged WT Hfe were utilised. The Y245X Tfr2 mutant used here has been characterised previously [18] and in humans the corresponding truncation mutation, Y250X, is associated with hereditary hemochromatosis (HH) type III [1]. Figure 1 shows the relative expression of Hfe, Tfr1 and Tfr2 in untransfected and transfected cells. We observed two bands for Tfr2 which could be due to a glycosylated form of the protein [19]. Figure 1B shows the truncated Tfr2^Y245X^ protein at approximately 40kDa. Tfr1 is endogenously expressed in Hepa1-6 cells. 

**Figure 2:**
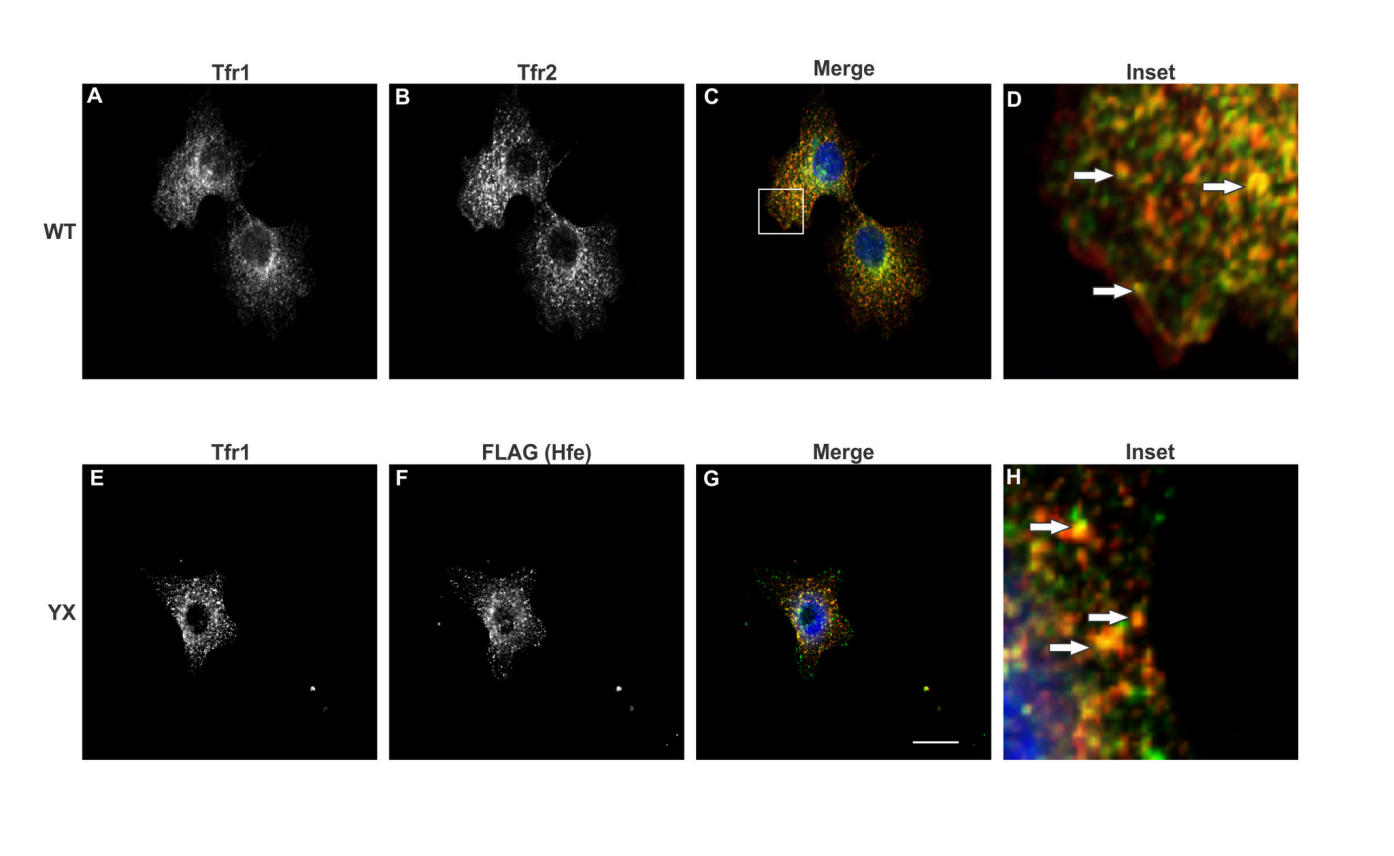
Co-localization of Tfr1 with Hfe and Tfr2. Confocal microscopy analysis of Tfr1, Tfr2 and Hfe in Hepa1-6 cells expressing either (A, B and C) myc-tagged Tfr2 alone or (E, F and G) FLAG-tagged Hfe alone. The transfected proteins were co-localized with endogenous Tfr1. Images were obtained using a Nikon C2 confocal microscope. The insets D and H show the co-localization represented by arrows. Scale bar= 20µm.

**Figure 3:**
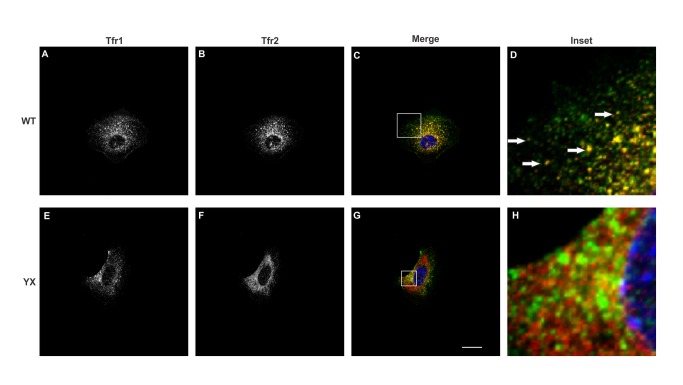
Co-localization of Tfr1 and Tfr2 in Hepa1-6 cells. Confocal microscopy analysis of Hepa1-6 cells stably co-expressing Hfe and either Tfr2 (WT) or truncated Tfr2^YX^ (YX). The localization of Tfr1 (A, E) appears to be endosomal as previously shown, the wild type Tfr2 (B) also localizes to an endosomal compartment and to the plasma membrane, represented by white arrows whereas the Tfr2^YX^ (F) appears to accumulate in ER (as previously described by our laboratory, [16]. Tfr2^YX^ does not co-localize with Tfr1 (H). Images were obtained using a Nikon C2 confocal microscope. Scale bar =20µm.

**Figure 4:**
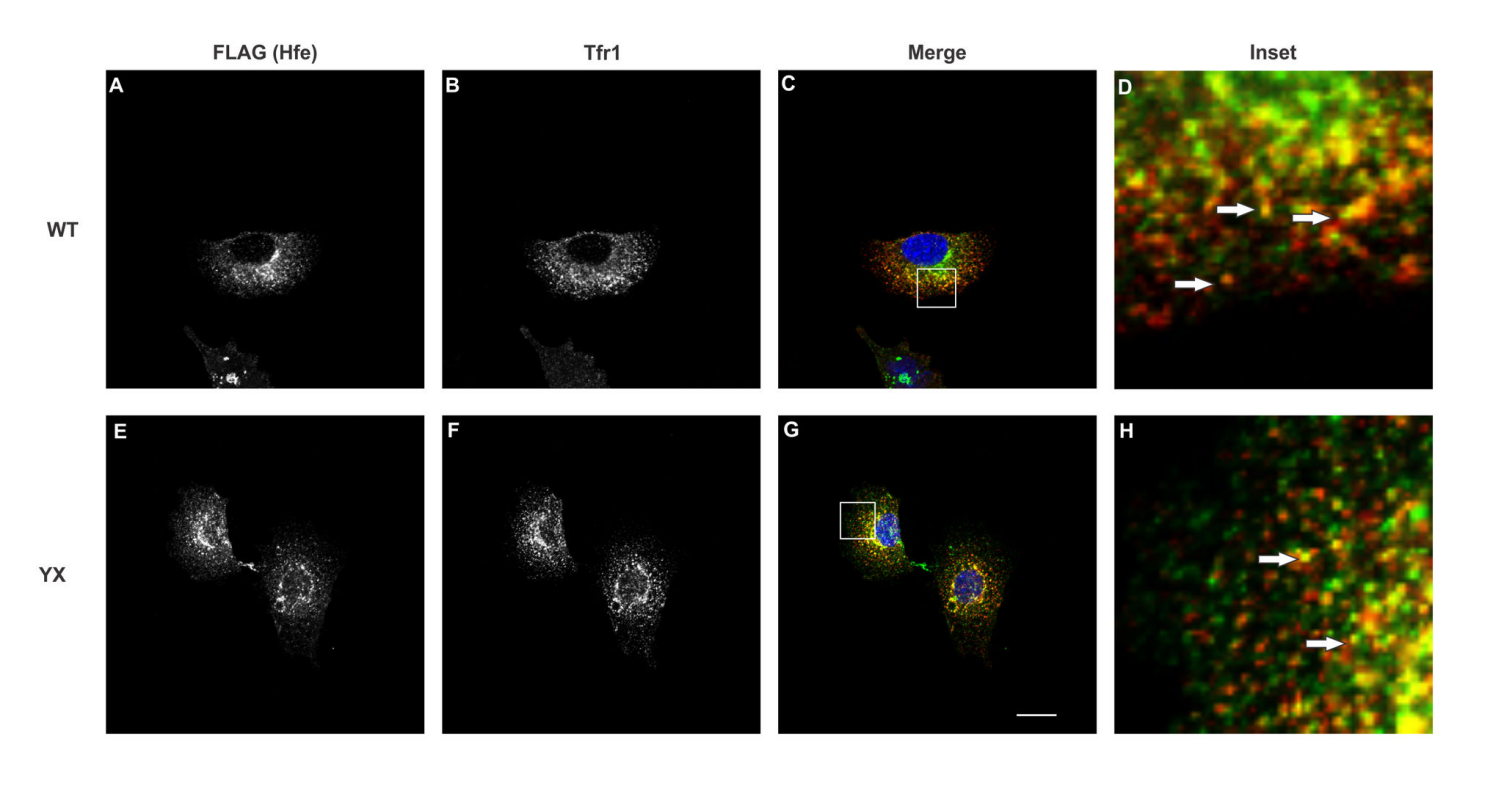
Co-localization of Hfe and Tfr1. Confocal analysis was performed to determine the co-localization of Hfe and Tfr1 in Hepa1-6 cells stably co-expressing Hfe and either Tfr2 (WT) or truncated Tfr2^YX^ (YX). Hfe (A and E) and Tfr1 (B, and F) co-localize in endosomal compartments and on the membrane (represented by white arrows) in the cells stably co-expressing Hfe and WT Tfr2 (C and G) or YX Tfr2 (D and H). The images were obtained using a Nikon C2 confocal microscope. Scale bar=20µm.

**Figure 5:**
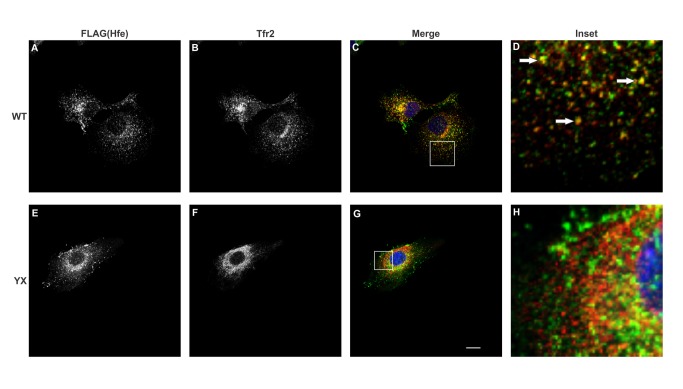
Co-localization between Hfe and Tfr2. Confocal microscopy analysis of Hepa1-6 cells stably co-expressing Hfe and either Tfr2 (WT) or truncated Tfr2^YX^ (YX) reveals that Hfe and Tfr2 do not co-localize significantly in cells stably co-expressing Hfe and WT Tfr2 (C and D) or truncated YX Tfr2 ( G and H). There is a small degree of overlap seen in some structures in WT (D) suggesting that Hfe and Tfr2 could be transiently present in the same sub-cellular structures. Images were acquired using Nikon C2 confocal microscope. Scale bar=20µm.

Overexpression of tagged proteins may lead to their aggregation [20] or mislocalization [21,22] thus affecting their function. In order to determine whether stable co-expression of Hfe and Tfr2 in Hepa1-6 cells affects their localization, we examined the localization patterns of these proteins in cells expressing either myc-tagged WT Tfr2 or FLAG-tagged WT Hfe singly or cells co-expressing both Hfe and Tfr2. The confocal images in Figures 2, 3, 4 and 5 show that both Hfe and Tfr2 have a similar localization whether expressed singly or together. This indicates that the co-expression of Hfe and Tfr2 does not affect their localization and function. FLAG-tagged Hfe, endogenous Tfr1 and myc tagged Tfr2 localized mostly intracellularly but expression of all three proteins on the plasma membrane was also detected as shown in Figures 2, 3, 4, and 5. 

Co-localization of two proteins suggests that they are present in the same subcellular compartments. In order to examine the co-localization between Hfe, Tfr1 and Tfr2 a double immunofluorescence experiment was performed. Panels 3C and 3D show that Tfr1 partially co-localizes with WT Tfr2. We had shown earlier that Tfr2^Y245X^ has a defect in trafficking and is retained in the endoplasmic reticulum [16]; panel 3F shows that there is a change in the localization of the truncated protein as compared to 3B. The change in localization of Tfr2^Y245X^ leads to a decrease in co-localization of Tfr1 and Tfr2 as seen in 3H. The co-localization signal could be observed intracellularly as well as on the surface of the cells.

In the cells expressing Hfe and WT Tfr2 and Tfr2^Y245X^, we observed a significant co-localization between Hfe and Tfr1 (Panel 4 C and D). 

It was reported previously that HFE and TFR2 co-localize intracellularly in the crypt cells of the duodenum [23]. In our co-expression system we observed nominal co-localization between Hfe and WT Tfr2 in punctuate intracellular vesicles (Panel 5 C and D). The minimal co-localization of the two proteins suggests that they could transiently co-exist in the same sub-cellular compartment and hence could be involved in an interaction. 

### b: *In situ* proximity ligation assay shows that Hfe and Tfr2 do not interact

Co-immunoprecipitation (Co-IP) is a standard method to determine whether two proteins of interest form a complex; however sometimes this technique fails to detect weak or transient interactions [24]. Co-IP involves lysis of the cells, this could bring proteins which are usually in different subcellular compartments together and result in false positive detection of an interaction [24]. The nominal co-localization observed between Hfe and Tfr2 in Figure 5 suggests that a small proportion of molecules may be involved in an interaction. A recently developed commercial assay (Duolink) based on proximity ligation (PLA) overcomes these limitations [25]. The advantage of PLA over conventional Co-IP is that it identifies individual interactions between two proteins in their native form that may be weak or transient in nature. The assay results in a fluorescent signal in the form of a spot when the two proteins of interest are closer than 40nm. To determine the interactions between Hfe, Tfr1 and Tfr2 using the PLA we used Hepa1-6 cells stably expressing Hfe and Tfr2.

**Figure 6:**
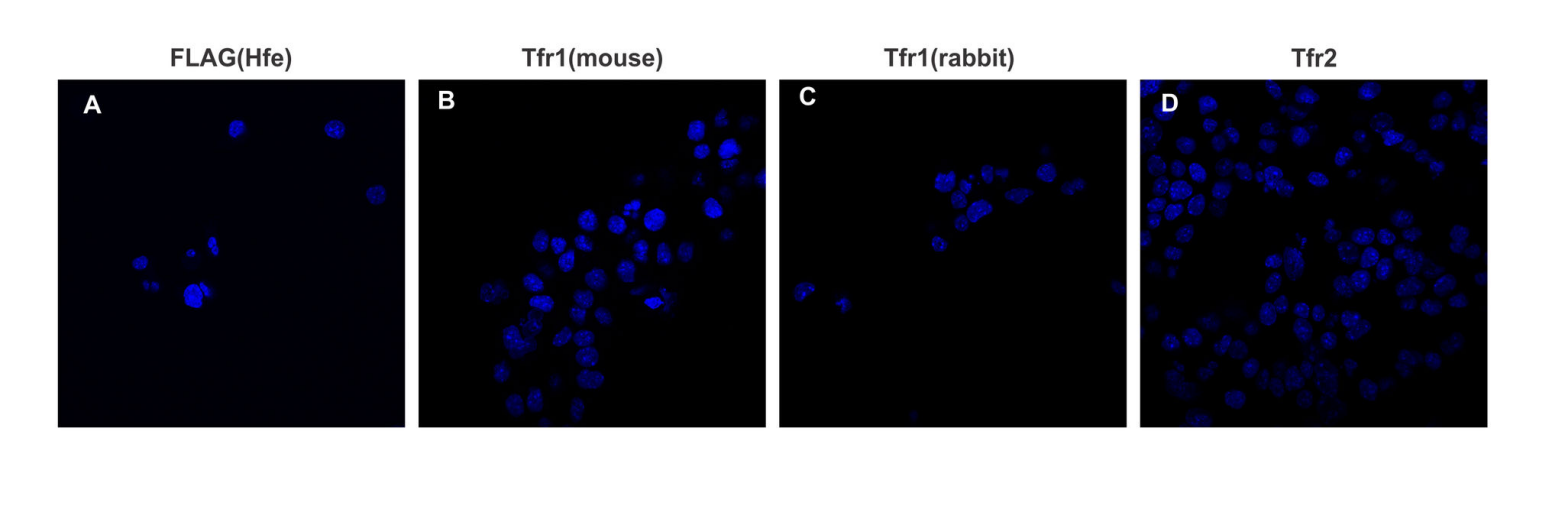
Specificity of PLA for transfected Hepa 1-6 cells. PLA was performed on Hepa1-6 cells stably co-expressing Hfe and Tfr2 (WT).The non-specific signal was examined by incubating Hepa1-6 cells with single antibodies directed against FLAG (A), Tfr1 (mouse) (B), Tfr1 (Rabbit) (C), or Tfr2 (D). The absence of any proximity signal indicates that the assay is specific. Tfr1 (mouse) – anti Tfr1 antibody raised in mouse, Tfr1 (Rabbit)- anti Tfr1 antibody raised in rabbit.

**Figure 7:**
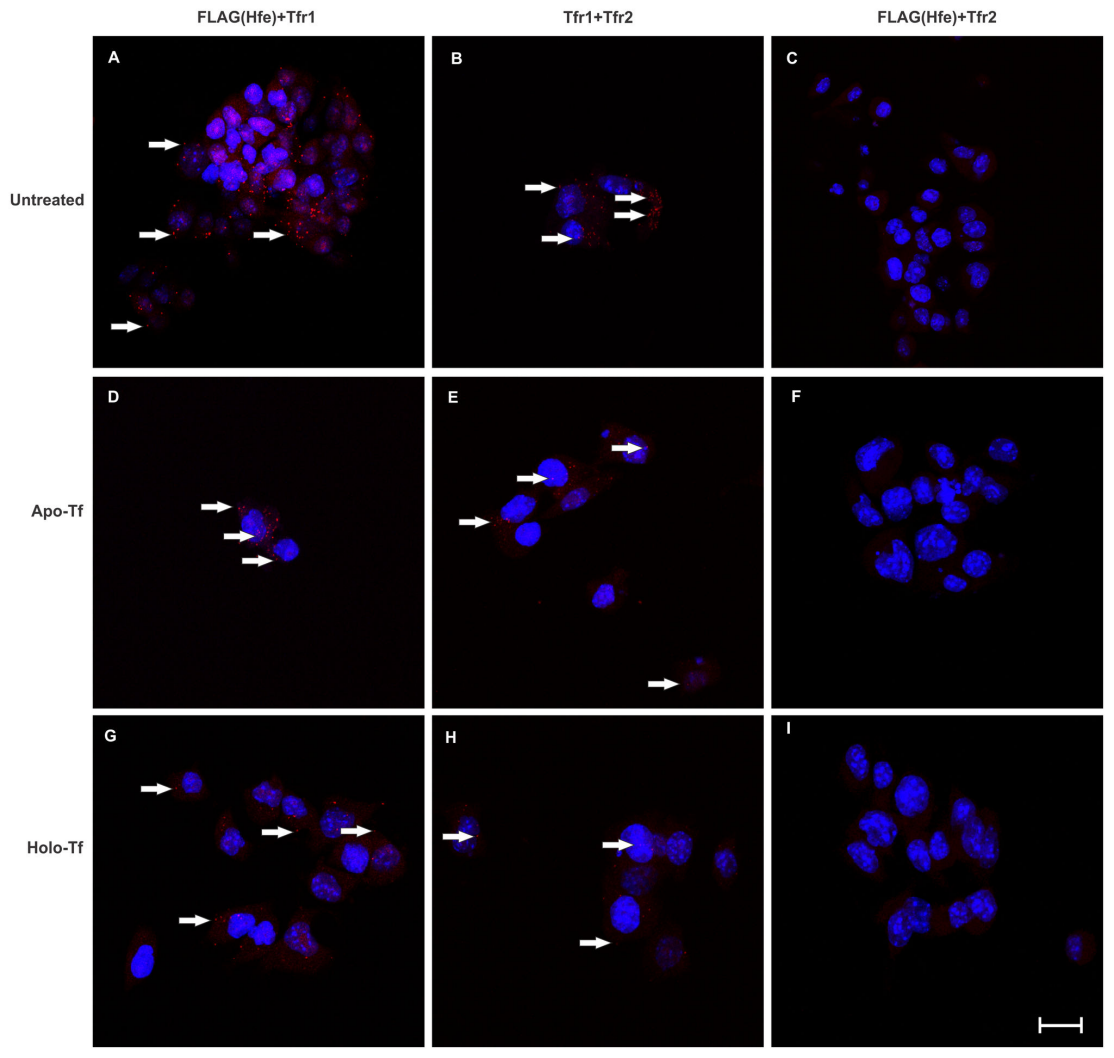
PLA shows that Hfe and Tfr2 do not interact. PLA was performed on Hepa1-6 cells stably co-expressing Hfe and Tfr2 (WT). Previously characterized interactions between Tfr1 and Tfr2 (A, D and G) and Hfe and Tfr1 (B, E and H) were detected. Hfe and Tfr2 do not interact in untreated WT cells (C) or cells treated with apo-Tf (F) or holo-Tf (I). The arrows indicate the red spots representative of the interactions between the proteins of interest, each red spot is equivalent of one molecular interaction. Images were acquired using a Nikon C1 confocal microscope. Scale Bar=20µm. Apo-Tf cells were cultured in 2mg/ml of apo-Tf for 24 hours and Holo-Tf cells were cultured in 2mg/ml of holo-Tf for 24 hours. FLAG+ Tfr1 represents the experiment where antibodies against FLAG tagged Hfe and Tfr1 (raised in Rabbit) were used, Similarly Tfr1+Tfr2 represents the experiment where Tfr1 (raised in mouse) and Tfr2 were used, and FLAG+Tfr2 represents the experiment where antibodies against FLAG tagged Hfe and Tfr2 were used.

The specificity of the assay was tested using a single antibody directed against FLAG, Tfr1 and Tfr2, these also served as negative controls (Figure 6 A, B, C, D). The absence of any spots indicates that the assay is specific with minimal non-specific proximity signals. The PLA was able to identify Tfr1 and Tfr2 as partners in Hepa1-6 cells, as indicated by the red dots in the panels (Figure 7 A); each red dot represents a molecular interaction between the two proteins of interest. This is in agreement with previously published data [26] where it was shown that Tfr1 and Tfr2 can form heterodimers. Similarly, Hfe and Tfr1 were also identified as proximity partners (Figure 7 B). The absence of any PLA signal in panel 7 C indicates that Hfe and Tfr2 do not interact in the Hepa 1-6 cells co-expressing Hfe and Tfr2.

The proposed iron sensing model suggests that in the presence of holo-Tf, HFE is released from TFR1 and made available to interact with TFR2. We did not observe an interaction between Hfe and Tfr2, suggesting that under normal conditions there is little or no interaction between Hfe and Tfr2. In order to determine the dynamics of Hfe and Tfr2 interaction in the presence of holo-Tf, we treated the cells expressing WT Hfe and Tfr2 with 2 mg/ml of holo-Tf or apo-Tf (as previously described [8]) for 24 hours; PLA was performed on these treated cells. The panels 7 D-F represent the cells treated with apo-Tf and panels 7 G-I represent the cells treated with holo-Tf. The results show that in the presence of apo- or holo-Tf, Tfr1 and Tfr2 form heterodimers (7 D and G, respectively), and Hfe and Tfr1 interact (7 E and H, respectively). The holo-Tf treatment resulted in a decrease in the number of red spots per nuclei (panel 7 G and H). It has been shown that in the presence of holo-Tf Tfr1 is internalized and the interaction between Hfe and Tfr1 decreases [10]. The absence of any proximity signals in the panels 7 F and I indicates that Hfe and Tfr2 do not interact in the presence of either apo- or holo-Tf in Hepa 1-6 cells. These results are contrary to the previously published data which suggests that in the presence of holo-Tf, the interaction between Hfe and Tfr2 increases. 

### c: Co-Immunoprecipitation experiments confirm the absence of interactions between Hfe and Tfr2

**Figure 8:**
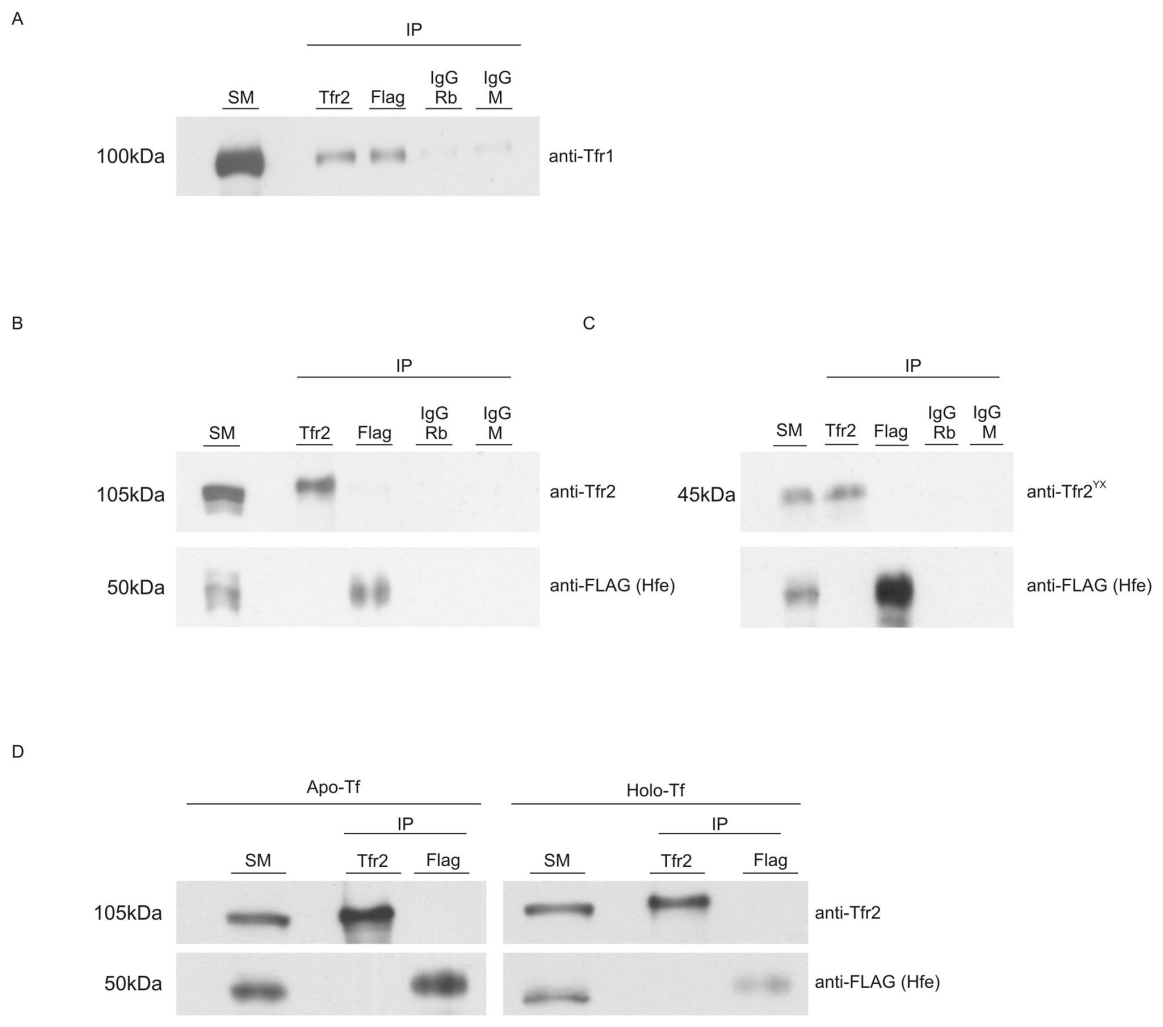
Co-immunoprecipitation experiments reveal that Hfe and Tfr2 do not form a complex. Hepa1-6 cells stably co-expressing FLAG-tagged Hfe and myc-tagged Tfr2 (WT) were used to determine interactions between Hfe, Tfr1 and Tfr2. (A) Tfr1 interacts with Hfe and Tfr2. A Co-IP with IgG rabbit and mouse antibody shows some non-specific binding with Tfr1. (B) Co-immunoprecipitation with anti-DYKDDDDK beads and anti-Tfr2 antibody reveals that Hfe and Tfr2 do not form a complex with each other. (C) Hfe and Tfr2 do not interact with each in Hepa 1-6 cells co-expressing FLAG-tagged Hfe and myc-tagged Tfr2^YX^. (D) WT cells were cultured in 2mg/ml apo- or holo-Tf for 24 hours and a Co-IP was performed with anti DYKDDDDK beads and anti Tfr2 antibody. Hfe and Tfr2 do not interact with each other in the presence of either apo- or holo-Tf. SM - starting material or input, IgG M - IgG mouse, IgG Rb - IgG Rabbit, Apo-Tf- apotransferrin, Holo-Tf- holotransferrin.

Since we did not detect any interaction between Hfe and Tfr2 using the highly sensitive PLA, we hypothesized that any interaction could be transient. To ensure that the weak and transient interactions were stabilized the cells were treated with Bissulphosuccinimidyl suberate (BS3) a surface crosslinker for 30 minutes at 4 °C before harvesting the cells for protein extraction. The lysates from Hepa 1-6 cells stably expressing Hfe and Tfr2 were used to precipitate proteins which form complexes with either Hfe (FLAG) or Tfr2. Rabbit IgG and mouse IgG were used as controls to detect any non-specific binding. Figure 8 A shows that Tfr1 can form a complex with Hfe and Tfr2 as previously characterized. These results were indicative that both the expressed proteins were functional. A small amount of non-specific binding of Tfr1 can be detected in the lanes representing rabbit IgG and mouse IgG IPs. Figure 8 B indicates that neither Hfe nor Tfr2 are present in a protein complex with each other in the Hepa 1-6 cells. The absence of any bands in the IgG lanes indicates that the assay is specific. 

In a previous study involving a domain swap of Tfr1 and Tfr2 [8] it was shown that amino acid residues 104-249 of TFR2 are sufficient for it to interact with HFE. We performed a Co-IP experiment with anti-FLAG and anti-Tfr2 using lysates from cells expressing Hfe and Tfr2^Y245X^; Figure 8 C shows that the truncated form of Tfr2 which contains only the first 244 amino acids of Tfr2 does not exist in a complex with FLAG-tagged Hfe.

A Co-IP performed on the lysates of cells treated with apo- and holo-Tf shows that Hfe and Tfr2 do not interact with each other in either of the conditions (8 D). These results are contrary to the previously published data which suggested that in the presence of holo-Tf, Hfe and Tfr2 form a complex. 

## Discussion

Previous studies using transient overexpression systems showed that HFE and TFR2 interact [8–10] and that this interaction is important for HFE and TFR2 mediated regulation of hepcidin [10,11]. Some recent studies have suggested that HFE and TFR2 can independently regulate hepcidin and could be acting in parallel to each other rather than synergistically [12–14]. 

To examine putative interactions between these proteins we developed a novel expression system where both Hfe and Tfr2 are stably co-expressed using a tricistronic vector. Using immunofluorescence and Co-IP we were able to show that a stable co-expression of Hfe and Tfr2 does not affect their localization or function. We observed partial co-localization between Tfr1 and Hfe, and Tfr1 and Tfr2 suggesting that these proteins could be present in similar subcellular compartments and hence could interact or form a complex. As previously described, we again showed that the YX mutant of Tfr2 (which contains only the first 244 amino acids of the protein) is retained in the endoplasmic reticulum [16]. 

The co-localization studies indicated some overlap between Hfe and Tfr2 in the cells expressing WT Hfe and Tfr2; hence we hypothesized that they could be involved in transient or weak interactions which sometimes cannot be detected using Co-IP. The PLA is a sensitive technique which can be used to visualize weak and transient interactions at a cellular and molecular level. A signal is observed only if the two proteins of interest are in close proximity with each other (40nm or less). This is the first report describing the interactions between Hfe-Tfr1 and Tfr1-Tfr2 at a cellular level. The images in Figure 7 suggest that there are more Tfr1-Tfr2 dimerisation events as compared to interactions between Hfe and Tfr1. Although previous studies have suggested that HFE and TFR2 interact, we did not detect any interactions between Hfe and Tfr2 in our stable expression system when using either the highly sensitive PLA or Co-IP. It was shown earlier that a chimera of TFR1 and TFR2 consisting of TFR2_104-250_ is able to interact with HFE [8]. A Co-IP experiment performed using cell lysates from our co-expression system shows that Tfr2^YX^ and Hfe do not form a complex with each other.

The iron sensing model suggests that in the presence of holo-Tf the interaction between HFE and TFR2 increases and this interaction signals a cascade of events which leads to HFE and TFR2 mediated regulation of hepcidin. We treated the Hepa1-6 cells stably co-expressing Hfe and WT Tfr2 with apo- or holo-Tf in order to determine whether Hfe and Tfr2 interact in our co-expression system. The results suggest that Hfe and Tfr2 do not form a complex with each other when they are stably co-expressed. The results from our experiments and recent studies [12,14] indicate that Hfe and Tfr2 can act independently and do not need to interact as earlier studies had suggested. Although previous in-vitro studies using transient expression systems have been able to detect the interactions [8–10], our results probably differ because of a stable co-expression of the two proteins. This is supported by a recent in-vivo study showing that mice expressing transgenic Hfe could regulate hepcidin independently of Tfr2 [12]. The authors did not observe any interaction between Hfe and Tfr2 and had suggested that some inhibitor in the tissue lysate could be responsible for degrading or inhibiting the complex. However, our results indicate that even in pure cell populations expressing the two proteins, Hfe and Tfr2 do not form a complex. One of the drawbacks of our system is that unlike some previously published studies [10] we could not measure the regulation of hepcidin in the presence of holo-Tf. 

 In addition to this, a large scale immunoprecipitation was performed in our laboratory using a previously characterized, highly specific Tfr2 antibody generated in the laboratory [17] and total liver homogenates (results not shown), to identify the protein partners that could interact with Tfr2. A mass spectrometric analysis of the immunoprecipitated complexes did not reveal Hfe as a binding partner of Tfr2. In the same experiment we were able to identify Tfr1 as a binding partner for Tfr2. 

The results from our studies combined with that of previous studies suggest independent roles for Hfe and Tfr2 in regulating hepcidin. Future studies should be directed at dissecting these roles and identifying whether Hfe and Tfr2 are a part of a single signalling cascade, or if they act in parallel to each other to regulate hepcidin.
